# Practical Notes and Formulæ

**Published:** 1885-06-20

**Authors:** 


### PRACTICAL NOTES AND FORMULÆ.

**Dysentery.**—The following, after a dose of castor oil, is an excellent remedy in dysentery, especially in children :

℞. Monobrominated camphor....................gr. ii
Pulv. ipecac.................................gr. iii
Muriate Morphine.............................gr. i

M. Triturate thoroughly and divide into twenty powders. Give one every three hours to a child one or two years old until the bowels are somewhat restrained, and then one after each action until relieved.

**Dysentery.**—

℞. Sulphate of soda..............................℥ i

Water...........................................℥ iv

Tinc. ipecac....................................ʒ ss

M. Dose for an adult one tablespoonful every hour until the discharges change, then in smaller doses for a few hours, after which add an opiate to the remedy, or check the bowels with morphine or dovers powder. During this treatment mustard should be applied to the abdomen, followed by warm fomentations.

**An Ohpthalmic Antiseptic Solution.**—In a recent discussion at the Paris *Academie de Medicine* (*Union Med.*) M. Pans stated that, in a large practice in ophthalmal surgery, he had never had any cases of erysipelas after the use of the following mixture :

℞. Bichloride of mercury, } aa................ 5 grains

Chloride of ammonium }

Glycerin.................................. 3 drachms

Water..................................... 3 quarts.

The parts are washed with the solution, after the operation a piece of linen dipped in the fluid is applied to the affected eye.—*N. Y. Medical Journal.*

**A Diuretic Mixture.**—Dr. Joseph Mullone, of Lyons, Ind., writes to us that he has treated a number of severe cases of anasarca, most of them of distinctly malarial origin, and has been much pleased with the action of the following formula:

℞. Compound spirit of juniper...............1 pint
Sulphate of iron.............................2 drachms
Acetate of potassium.........................½ ounce
Fluid extract of digitalis...................2 fluidrachms
Syrup of squill..............................½ fluidounce.

Dose, a tablespoonful three times a day. In severe cases the patient is to drink also a cold infusion of elder root.—*New York Medical Journal.*

**Oleate of Bismuth in Eczema.**—Van Harlingen, in *Philadelphia Medical Times:*

℞. Bismuth oxid.................................ʒi

Acidi oleici.................................℥i

Ciræ albæ.................................℈iii

Vaseline.................................ʒix

Ol rosæ.................................m ii.

M.

Its action in eczema of the hands is particularly satisfactory.—*Philadelphia Medical Times.*

**Wizard Oil.**—The American Druggist asks for the formula of Hamlin's Wizard Oil. The following is said to closely resemble this preparation :

℞. Spts. camphor.................................℥ 10

Water of ammonia.................................℥ 5

Oil of sassafras.................................℥ 5

Oil of cloves.................................℥ 2

Oil of turpentine.................................℥ 5

Chloroform.................................℥ 1

Alcohol.................................℥ 20

Mix.—*National. Druggist*

**Black Draught of British Pharmacopea an Excellent Purgative.**—

℞. Infus. sennæ.................................14 parts

Magnesiæ sulp.................................4 parts

Ext. glycyrrhizæ.................................½ part

Tinct. sennæ.................................2½ parts

Tinct. cardamon: co.................................1¼ parts

M. Dose ℥ j. to ℥ ss.

**Cough Remedy.**—The Weekly Drug News recommends the following as a good remedy for cough without opium (which is often objectionable). This preparation is especially valuable in irritating and obstinate coughs, and is a pleasant sedative and expectorant cough remedy :

℞. Bromide potassium.................................1 ℥ av.

Tincture sanguinaria (blood root).................................3 fl. ʒ

Tinture of hyoscyamus.................................2 fl. ℥

Ether (sulphuric).................................½ fl. ℥

Syrup of ipecac.................................2 fl. ℥

Syrup of tolu.................................7 fl. ℥

Alcohol.................................1 fl. ℥

Water.................................3 fl. ℥

Dissolve the bromide of potassium in the water and mix the solution with the syrups. Mix the alcohol with the ether and tinctures, then add the mixtures to the syrups and mix.

Dose, the same as other cough remedies, (1 ʒ)but may be given freely without injury.—*Louisville Med. News.*

